# High selfing rate and inbreeding depression correlate with past range expansion in an endangered African timber species *Pericopsis elata*

**DOI:** 10.1038/s41437-026-00845-6

**Published:** 2026-04-23

**Authors:** Bloude L. B. Toumba-Paka, Gaël U. D. Bouka, Surabhi Ranavat, Dieu-Merci Assumani, Saskia Sergeant, Raissa Kouamo, Bienvenu S. Ango, Freddy D. M. Mbika, Bonaventure Sonké, Joël Loumeto, Germain Mbock, Olivier J. Hardy

**Affiliations:** 1 Evolutionary Biology and Ecology, Faculté des Sciences, Université libre de Bruxelles (ULB), Brussels, Belgium; 2 Laboratory of Biodiversity and Ecosystems and Environmental Management, Faculty of Science and Technology, Marien Ngouabi University, Brazzaville, Republic of the Congo; 3 Faculté de Gestion des Ressources Naturelles Renouvelables, Université de Kisangani, Kisangani, Democratic Republic of the Congo; 4 Institut National pour l’Etude et la Recherche Agronomiques (INERA – Yangambi), Yangambi, Democratic Republic of the Congo; 5 Cirad, UMR AGAP Institut, Petit Bourg, Guadeloupe France; 6 University of Yaoundé I, Plant Systematic and Ecology Laboratory, Yaoundé, Cameroon; 7 Ecole Nationale des Eaux et Forêts de Mbalmayo, Ministère Des Forêts Et De La Faune, Mbalmayo, Cameroon

**Keywords:** Inbreeding, Consanguinity

## Abstract

Self-fertilization and inbreeding depression (δ) are expected to co-evolve, particularly during range expansion, yet few studies have examined this dynamic in tropical trees, which could be relevant for their conservation. We investigated the mating system of *Pericopsis elata* (Fabaceae), an endangered timber species with a fragmented distribution in Central Africa comprising two distinct genetic clusters: the Sangha River Interval (SRI) and the eastern cluster (Congolia). The SRI has recently expanded its range. We hypothesized that this expansion influenced both selfing rates and δ. Using microsatellite markers, we genotyped 54 mother trees and their offspring across five populations. Results revealed exceptionally high selfing rates in the SRI (mean = 0.88), significantly exceeding those in the eastern cluster (0.54). Within the SRI, selfing rates were consistent (0.84–0.92), while δ remained high (0.82–0.97), except at the expansion front (0.37). These findings suggest that past range contraction and fragmentation may have favored selfing for reproductive assurance, albeit without further increasing selfing rates during the recent expansion. However, the persistence of strong δ despite high selfing rates challenges classical expectations of purging deleterious alleles. This study underscores the need to consider local variation in mating systems when developing conservation strategies for *P. elata*. More broadly, it provides rare empirical evidence of intraspecific mating system variation in tropical trees with high selfing rates without necessarily reducing δ. From an applied perspective, plantations of *P. elata* would benefit from outcrossed seeds, though seed number and position within pods were not correlated with outcrossing rate.

## Introduction

Most flowering plant species are hermaphroditic, enabling self-fertilization (selfing) in self-compatible species, leading to a wide range of mating strategies, spanning from highly outcrossing to predominantly selfing species (Barrett and Eckert 1990; Goodwillie et al. 2005; Schemske and Lande 1985). Some evolutionary models predict that selfing rate, inbreeding depression, and genetic load evolve together toward 0% or 100% selfing (Charlesworth et al. 1990; Schemske and Lande 1985). However, mixed-mating systems are the most common among animal-pollinated species (Goodwillie et al. 2005; Whitehead et al. 2018), raising important questions regarding their evolutionary significance. Are these systems adaptive strategies that maximize reproductive success, or do they represent transitional phases toward self-fertilization (Schemske and Lande 1985; Stebbins 1957; Winn et al. 2011)? Although much research has explored mating system variation among species, studies also suggest significant variation among conspecific populations (Barrett 2002; Barrett and Eckert 1990; Crawford et al. 2022; Whitehead et al. 2018). Population variation allows us to uncover how ecological and genetic factors drive shifts in mating strategies.

The study of population-level mating systems is particularly relevant for understanding plant evolution and conservation in tropical regions. Tropical tree species often exhibit mechanisms that prevent self-fertilization and promote outcrossing (Dick et al. 2008; Goodwillie et al. 2005; Ward et al. 2005). However, exceptions occur, as revealed by recent observations on *Pericopsis elata* (Fabaceae), a tropical African rainforest tree species, exhibiting a mixed-mating system with an estimated mean primary selfing rate of 54% (Angbonda et al. 2021). *Pericopsis elata* is distributed in fragmented and differentiated populations across Central and West Africa (Bourland et al. 2012a; Piñeiro et al. 2021; Ranavat et al. 2025). In Central Africa, two main genetic clusters occur and differ in genetic diversity (Micheneau et al. 2011; Ranavat et al. 2025). The eastern genetic cluster, located in northeastern Democratic Republic of Congo in the Congolian phytogeographic region, is characterized by higher genetic diversity, while the western genetic cluster, spanning the northwest of the Republic of Congo and southeast of Cameroon, in a phytogeographic area called the Sangha River Interval (SRI) (Gillet and Doucet 2012), exhibits reduced genetic diversity. Moreover, the SRI genetic cluster displays a decay of genetic diversity from east to west, consistent with a hypothesis of recent range expansion from a refuge population situated in the northern Republic of Congo, possibly after the last glacial maximum (Ranavat et al. 2025). This expansion is believed to have involved serial founder events, which led to the erosion of genetic diversity in newly established populations (Piñeiro et al. 2021; Ranavat et al. 2025).

Despite the documented genetic diversity differences between *P. elata* populations, its mating system has only been studied in the eastern Central African genetic cluster (Angbonda et al. 2021, 2024). Little is known about the mating system dynamics in populations that have experienced recent expansion. Studying these dynamics is crucial for understanding evolutionary changes in mating strategies during population establishment. During such events, species often encounter mate limitations and new environmental conditions within colonized regions (Barrett and Harder 2017; Stebbins 1957). These conditions can favor the evolution of selfing, as self-fertilization provides reproductive assurance under circumstances where mates or pollinators are scarce (Barrett and Eckert 1990; Barrett and Harder 2017; Pannell 2016). Understanding the degree of selfing and outcrossing in *P. elata* populations could shed light on the factors driving changes in mating systems and offer insights into how plants adapt to fragmented landscapes. Furthermore, these findings could have significant implications for the conservation of *P. elata*, which is classified as an endangered species due to its former overexploitation in West Africa (Bourland et al. 2012b), as mating systems play a critical role in populations’ ability to adapt to environmental changes (Barrett and Harder 2017; Glémin and Ronfort 2013; Hartfield et al. 2017; Hodgins and Yeaman 2019).

One expected consequence of self-fertilization is inbreeding depression (ID), which arises from increased homozygosity in inbred individuals, leading to the expression of deleterious recessive alleles or disrupting the fitness advantage conferred by heterozygosity at overdominant loci (Charlesworth and Charlesworth 1987; Charlesworth and Willis 2009; Lloyd 1979). Reduced fitness in selfed individuals compared to outcrossed individuals has been documented in tropical trees (Hufford and Hamrick 2003; Ismail et al. 2014; Naito et al. 2005; Rymer et al. 2015; Takeuchi et al. 2020), including in *P. elata* (Angbonda et al. 2024; Ngongo et al. 2025), and ID tends to be much stronger in species with long generation time (Duminil et al. 2009). The extent of ID within a population is influenced by its genetic load at polymorphic loci, which evolves over time through selection against deleterious alleles or their fixation via genetic drift (Crnokrak and Barrett 2002). Populations that are highly inbred may experience lower levels of inbreeding depression due to either purging of the genetic load or its fixation (Barrett and Charlesworth 1991; Glémin 2003). If part of the ID results from overdominant loci, only strong genetic drift can lead to the fixation of an allele, reducing ID. However, fragmented populations, such as those of *P. elata*, may exhibit varying levels of inbreeding depression depending on their history and genetic structure. Given the contrasted patterns of genetic diversity and demographic history of the Central African *P. elata* clusters (Ranavat et al. 2025), investigating the evolution of inbreeding depression in the SRI populations of *P. elata*, as well as comparing these findings with eastern populations, could provide critical insights into the dynamics of mating systems within the species. These studies are essential for informing conservation and management strategies aimed at ensuring the long-term survival and genetic resilience of this endangered tropical tree.

Additionally, although previous studies highlight the advantages of outbred individuals in terms of growth dynamics (Angbonda et al. 2024; Ngongo et al. 2025), in light of the significant inbreeding depression observed in this species, the practical application of this knowledge remains limited. While selecting outcrossed seeds has been recommended to enhance plantation performance (Angbonda et al. 2024), identifying such seeds requires molecular analysis and specialized expertise. These laboratory-based approaches, though precise, are resource-intensive and thus impractical for routine use in large-scale or conventional reforestation efforts. Hence, a correlation between a phenotypic feature and the probability of outcrossing could be of great practical interest to select outbred seeds. Variability in seed number per pod within a plant may be directly influenced by pollination conditions, whether the pod resulted from selfing or outcrossing (Bradner and Frakes 1964; Pedersen 1968). Selfing often leads to fewer seeds per pod, likely due to partial seed abortion, reduced fertilization success, and lower pollen viability compared to outcrossing (Pedersen 1968). Moreover, in some species, differences in pollen-tube growth between self and outcross pollen have been observed (Aizen et al. 1990 for *Dianthus chinensis*, Figueroa-Castro and Holtsford 2009 for *Nicotiana longiflora*, Smith-Huerta 1996 for *Clarkia tembloriensis*, Yacine and Bouras 1997 for *Quercus ilex*). Such differences could affect fertilization success along the ovary axis, increasing the proportion of either outcrossed or selfed seeds at the stylar end or near the pod base and producing a spatial gradient of outcrossing rates within individual pods (Cooper and Brink 1940; Horovitz et al. 1976). Therefore, both the number of seeds per pod and the position of the seed within a pod may serve as a proxy for identifying outcrossed seeds.

The following objectives were pursued here: (i) to assess changes in population genetic diversity along a westward range gradient and test for historical bottleneck signatures; (ii) to estimate individual and population selfing rates across Central Africa and test whether selfing rate changes with the expansion; (iii) to quantify lifetime inbreeding depression (ID) from the seedling stage to the adult stage for each provenance; and (iv) to test the influence of selfing rate on the number of seeds per pod and the position of seeds within a pod. To address these objectives, we formulated the following hypotheses: (H1) genetic diversity decreases and bottleneck signatures increase toward the western edge of the range as a result of multiple founder events; (H2) selfing rate increases in recently established populations due to mate or pollinator limitation during expansion; (H3) inbreeding depression is reduced in recently established populations because frequent selfing may have led to the purging of deleterious alleles; and (H4) the selfing rate varies according to seed number per pod and/or seed position within multiseeded pods.

## Methods

### Study species

*Pericopsis elata* is a large tree species belonging to the Fabaceae (Leguminosae) family (subfamily Papilionoideae), naturally occurring in fragmented populations from West Africa to the Congo Basin (Bourland et al. 2012a; Fayolle et al. 2015). It is a gregarious, light-demanding species that can grow up to 50 meters in height and 1.70 meters in diameter (Boyemba 2011). The species is fully self-compatible (Angbonda et al. 2021), with hermaphroditic flowers measuring approximately 15 mm in length and 13–14 mm in width (Boyemba 2011). Although its pollination biology has not been documented, pollination is likely performed by insects. The flowers produce flat, thin pod fruits, ranging from 9 to 17 cm in length and 2.5 to 3 cm in width (Bourland et al. 2012b). Pods are green when immature, turning brownish upon ripening, and are dispersed by wind, usually within 100 meters from the mother tree but long-distance dispersal also occurs occasionally (Angbonda et al. 2021). Each pod typically contains 1 to 4 seeds (Dickson et al. 2005). The seeds are reddish-brown, almost rectangular, and approximately 15 mm wide (Toussaint et al. 1953). The wood of *P. elata* is of exceptional quality, with diverse applications (Bourland et al. 2012b) and among the highest commercial values in the global tropical timber market (Bourland et al. 2012a). Overexploitation because of high demand for its wood and poor natural regeneration has led to a decline in natural populations, particularly in West Africa (Boyemba 2011). Consequently, the species has been listed in Appendix II of the CITES Convention since 1992 (Dickson et al. 2005).

### Study site and sampling

**Fig. 1:**
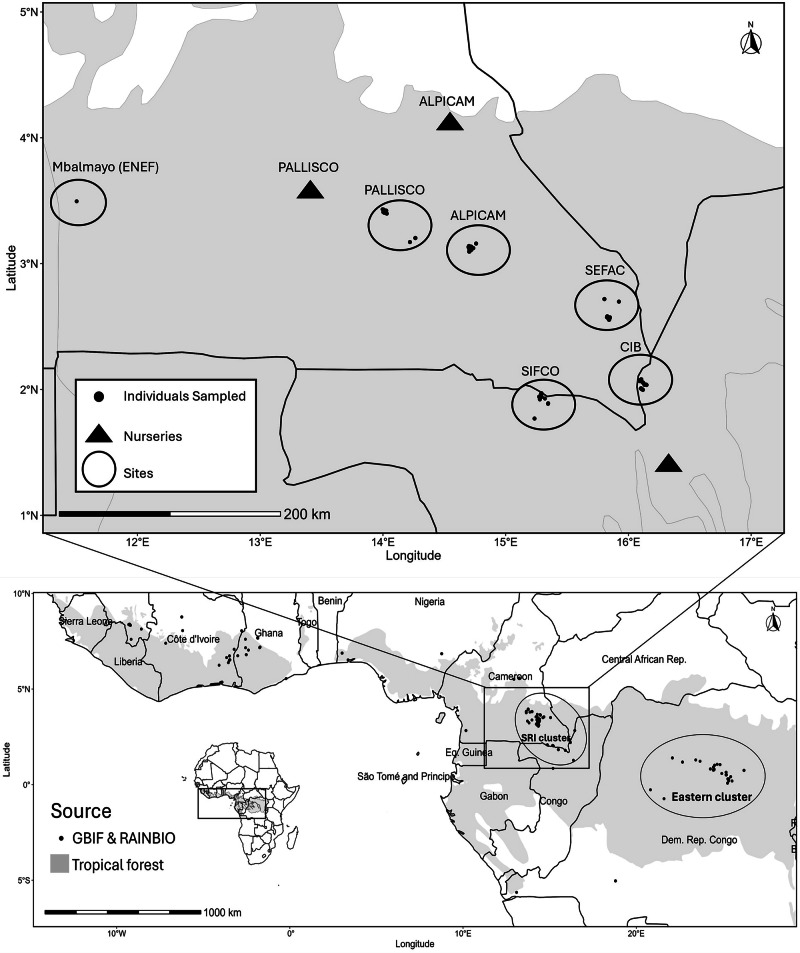
Study area (upper panel) and distribution (lower panel) of Pericopsis elata (black dots) in tropical African forests (gray area) according to GBIF and RAINBIO (Dauby et al. 2016) databases. The location of the nurseries where seedlings were raised, and of the ENEF garden where seeds from planted trees were used, is also shown.

Seed sampling was conducted in forest logging concessions from Cameroon and the Republic of Congo between February and April 2022 across five provenances, hereafter called Pa, Al, Se, Si, and Ci (from West to East), the initials of the logging companies managing the concessions (Fig. 1; Table 1). These sites were selected based on the local abundance of *P. elata* and to align with the species’ recent westward range expansion pathway throughout the SRI cluster (Ranavat et al. 2025). For each provenance, 100–200 seeds per tree were collected based on the availability of pods beneath the randomly selected seed trees. A minimum of 20 seed trees were sampled per provenance, except for Pa. Pods were gathered directly from beneath each tree, within a maximum radius of 10 m, to ensure accurate identification, with the assumption that the fruits were produced by the corresponding tree. Consecutive seed trees were separated by at least 100 m to minimize spatial overlap of families, and the maximal distance between trees from the same provenance ranged from 9.1 km to 22.5 km, except in Pa where the sampling occurred at the end of the fruiting season and the paucity of fruiting trees forced us to search 2 additional seed trees c. 35 km from the initial target site where only 11 seed trees could be sampled. In total, 98 seed families were obtained, ensuring robust representation of genetic variation across the species’ distribution range. In addition, for each seed tree as well as for surrounding *P. elata* adult trees (dbh ≥ 30 cm) encountered in the field, the diameter at breast height (DBH) and GPS coordinates were recorded, and a cambium sample was collected and dried in silica gel to preserve its DNA.

**Table 1:** Characteristics of five populations of Pericopsis elata from the western (SRI) gene pool, ordered from East to West, with the total number of sampled adult trees and the number of seed trees below which seed families were collected.

Provenance	Code	Geographical origin	Mean longitude	Mean latitude	Number of trees	Number of seed trees	Min-Max distance between trees (km)
CIB	Ci	UFA Kabo, Congo	16.113	2.038	70	24	0.007–9.107
SEFAC	Se	UFA 10009, UFA 10010, UFA 10012, Cameroon	15.836	2.603	34	20	0.004–18.122
SIFCO	Si	UFA Tala-Tala, Congo	15.293	1.928	31	20	0.029–22.497
ALPICAM	Al	UFA 10023, Cameroon	14.713	3.126	67	21	0.002–9.380
PALLISCO	Pa	UFA 10030, UFA 10039, Cameroon	14.103	3.337	48	13	0.002–38.601

Sampled pods were opened in the field to extract seeds, which were individually stored in paper envelopes, one per family, to reduce moisture retention and prevent rotting. To maintain germination potential, seeds were stored in a refrigerator as soon as possible until sowing. Seed sowing was conducted in nurseries provided by the logging forest companies Alpicam, Pallisco, and CIB between May and June 2022, after dividing each seed family lot into three. For each maternal plant and in each nursery, 40–50 seeds were sown in 2-liter polyethylene bags filled with soil collected from forests near the nursery. In some batches, one seed was planted per bag, while in others, up to three seeds were sown per bag. When multiple seeds germinated in the same bag, the seedlings were transplanted to distinct bags. Out of 98 families, we selected 60 that delivered enough seedlings to set up ongoing common garden progeny and provenance trials for growth monitoring. For the present study, we used these 60 families to assess their respective selfing rates. Before transplanting the seedlings into common garden plantations, one leaflet per seedling was harvested and dried in silica gel to preserve DNA.

To assess whether the number of seeds in pods or the seed position within a pod could predict whether a seedling resulted from outcrossing or selfing, we collected pods with varying seed counts from four *P. elata* trees planted in the garden of the National School for Water and Forest of Cameroon (ENEF), in Mbalmayo, in February 2024. These trees were relatively low in height, which allowed us to directly access ripe fruits still on the branches, rather than collecting fallen fruits beneath the trees. This ensured that the fruits were definitely from the maternal plant, and pods were selected based on the number of seeds they contained (1 to 4), rather than relying on fallen fruits. The cambium of maternal trees was collected as well. The ENEF garden contains at least 20 other planted adult *P. elata* trees, so we do not expect a lack of pollen donors.

### DNA extraction and genotyping

DNA was extracted from field-collected cambium (25–30 mg) of 257 adult trees across the 5 provenance populations, including the 60 mother trees used for the provenance trials, and the four ENEF seed trees, and from dry leaflets (15–25 mg) of 20 offspring for each of the 60 families. For seeds collected on the four ENEF trees, DNA was extracted from 96 seeds from one-seeded pods (24 for each mother tree), and 94 seeds from four-seeded pods using the two seeds at extreme position with respect to ovary from 47 pods (19 pods from each of two mother trees, 6 and 3 for the two other mother trees that produced few four-seeded pods). All extractions were performed using the NucleoSpin® 96 Plant II extraction kit (Macherey-Nagel, Dürren, Germany).

All samples were then genotyped at nine newly developed microsatellite loci (Tables S1 and S2), because the microsatellites previously developed by Micheneau et al. (2011) were not polymorphic enough in the SRI genetic cluster to discriminate between selfed and outcrossed individuals (Ranavat et al. 2025). These new microsatellites were designed using an unpublished draft genome of *P. elata*, originating from the Pa population, selecting microsatellites with at least 30 repetitions of dinucleotide motifs, favoring markers with high mutation rates. Multiplex polymerase chain reactions (PCR) were performed in a total volume of 15 µl on a Professional Basic thermocycler (Biometra, Göttingen, Germany). Conditions for the amplification and detailed primer descriptions are found in online supplementary material S1. PCR products were analyzed on a 3730 ABI 48-capillary DNA analyzer (Applied Biosystems) using 1 µl of PCR product, 12 µl of Hi-Di formamide (Life Technologies), and 0.2 µl of size marker (GeneScan 500 LIZ Size Standard, Applied Biosystems). Finally, genotypes were successfully obtained from a total of 1195 seedlings (15–20 per mother tree), 246 adults (mother trees and surrounding adults), and 144 seeds from the ENEF garden.

### Genetic diversity parameters and Bottleneck test

Standardized allelic richness, observed heterozygosity (*H₀*), unbiased expected heterozygosity (*Hₑ*), and inbreeding coefficients (*Fᵢ*) were calculated for both the seedling and adult generations, as well as for each respective provenance, using SPAGeDi v.1.5d (Hardy and Vekemans 2002). The genetic differentiation *(F*_ST_*)* between provenance pairs was estimated using SPAGeDi v.1.5d. The inbreeding coefficient was also computed more accurately using the Bayesian method implemented in INEST 2.2 (Chybicki and Burczyk 2009) with the full model, which estimates simultaneously inbreeding, null allele frequencies, and genotyping failure rates. This was done at the level of each provenance population and for the seeds from the ENEF garden. Paired *t*-test, considering each locus as a replicate, was used to assess the difference between cohorts (adults vs seedlings) in each provenance population. Finally, INEST 2.2 was also used to estimate the inbreeding coefficient of each individual.

To test for a bottleneck in *P. elata* populations, we used the M-ratio method (Garza and Williamson 2001) implemented in INEST 2.2. M-ratio represents the ratio between the number of alleles and the range of microsatellite allele sizes at a given locus. This approach compared the observed M-ratio values with expected values generated from coalescent simulations under the assumption of constant population size, using the two-phase mutation model (TPM) with default parameters. Statistical significance was assessed using a Z-test based on combined Z-scores.

### Selfing rate assessment

Paternity analyses were conducted to distinguish offspring originating from self-fertilization and cross-fertilization. To this end, we used the neighborhood model, implemented in the NMπ software (Chybicki and Burczyk 2010), known for its high accuracy when sufficiently polymorphic markers are utilized. The model used the genotypes of adults and seedlings along with their spatial coordinates to infer the likelihood of being selfed or outcrossed, while simultaneously estimating the genotyping error rate at each locus, the selfing rate, the pollen immigration rate, and two parameters of a power exponential pollen dispersal kernel. The model was applied separately on each provenance population to avoid possible biases due to population differentiation. Among the 1195 genotyped seedlings for 9 markers, only those with at least six successfully genotyped loci for both the seedlings and their mother were retained. Seedlings showing more than two loci without shared alleles with their mother were excluded. Additionally, for seedlings with exactly six genotyped loci, only one locus could lack a maternal allele. Consequently, 1004 seedlings met these criteria. The NMπ algorithm assigned the paternity of 952 of these seedlings with a probability higher than 0.8; 29 seedlings identified as selfed by NMπ analysis showed more than two mismatches with the mother tree, each involving differences greater than two base pairs. Given the doubt about their true maternity, these seedlings were excluded from further analysis. Ultimately, 922 assigned seedlings were retained. Additionally, only seed trees (54) with at least ten retained seedlings were considered for selfing rate assessment. We then estimated the selfing rate for each mother tree as the proportion of selfed seedlings. We also estimated the selfing rate separately for each provenance as the mean of the selfing rate of all seed trees retained within that provenance. We ended up with 82 progenies for which paternity analysis could not robustly classify as either selfed or outcrossed, so they were first excluded from our selfing rate estimations. However, one possible explanation for this lack of statistical power is that these progenies may have resulted from matings between related parents. Therefore, we also estimated the selfing rate by treating these progenies as outcrossed, providing a lower boundary.

A χ^2^ test on the numbers of selfed and outcrossed seedlings per family allowed us to assess whether maternal trees differ significantly in selfing rate. A Kruskal-Wallis test on the proportions of selfed seedlings per family allowed us to assess whether the selfing rate varies among provenance populations. We then assessed whether variation in selfing rates among seed trees could be explained by tree DBH, longitudinal coordinates, local density of adults at a radius of 100 m around the mother tree, and the heterozygosity of the mother tree, using generalized linear regression with a binomial family, implemented in the glm() function of the R statistical software. Using the genotyped seeds collected in the ENEF garden, we compared selfing rates between one-seeded pods and multiple-seeded pods, as well as differences between seeds from the extremities of the ovary within multiple-seeded pods, using χ^2^ tests.

### Inbreeding depression

An approximate Bayesian computation (ABC) framework (Csilléry et al. 2010) was developed to estimate the proportion of selfed adults and lifetime inbreeding depression (ID) in each provenance population, and in a population (“Biaro”) from the Democratic Republic of Congo (eastern cluster) using the data of Angbonda et al. (2021, 2024). The principle of the approach, coded in R language (v4.3.2), is to simulate differential mortality between selfed and outcrossed seedlings due to ID and identify under which ID levels we eventually reach the level of inbreeding in seedlings equal to the one observed in adults. ID is here defined as δ = 1 – *SRs* / *SRo*, where *SRs* and *SRo* are the survival rates until adulthood of selfed and outcrossed seedlings, respectively. To this end, for each population separately, individual estimates of inbreeding coefficients provided by INEST 2.2, *F*_*ic*_, were subdivided into adults and seedling cohorts, and for the latter into selfed and outcrossed seedlings. A simulation run consisted in (i) generating a random δ value from a [0, 1] uniform distribution, (ii) eliminating each selfed seedling with a probability equal to δ, (iii) among the outcrossed and surviving selfed seedlings, selecting a number equal to the number of adults available randomly, and computing the mean *F*_*ic*_ of these seedlings. A simulation run was retained if the absolute difference between the mean *F*_*ic*_ of the selected seedling and the observed mean *F*_*ic*_ of adults was less than 0.01. We ran 100,000 simulation runs for each provenance, and from the retained runs, we estimated the mean and 95% posterior distribution of the δ values and of the proportions of selfed seedlings eventually kept. Additionally, we tested for each population if the level of ID departed significantly from δ = 0 or 1 by running the same algorithm with 100,000 simulation runs but checking this time if the *F*_*ic*_ of selected seedlings was higher or lower than the mean *F*_*ic*_ of adults. Specifically, if more than 5% of simulations under δ = 0 resulted in mean seedling *F*_*ic*_ lower than mean adult *F*_*ic*_, we considered that there was no significant ID, and the lower bound for δ estimates was set to 0. Conversely, if more than 5% of simulations under δ = 1 resulted in mean seedling *F*_*ic*_ higher than mean adult *F*_*ic*_, we considered that the upper bound for δ estimates was 1.

### Research ethics

The research was conducted in compliance with current regulations.

## Results

### Patterns of genetic diversity, inbreeding, and bottleneck across a westward range

The nine newly developed loci showed much more polymorphism than the ones developed by Micheneau et al. (2011): the number of alleles per locus ranged from 21 (P91) to 29 (P92 and P96), with an overall average of 26.5 across all populations, while the observed heterozygosity (*H*_*o*_) and expected heterozygosity (*H*_*e*_) per locus ranged from 0.428 to 0.544 and from 0.8153 to 0.9636, respectively (Table S2).

While the average expected heterozygosity of adults (*H*_*e*_) did not differ significantly between populations (ranging from 0.808 to 0.897), observed heterozygosity (*H*_*o*_), standardized allelic richness (*A*_*R*_), and inbreeding coefficient (*F*_*ic*_) varied significantly (Table 2). The westernmost population, Pa, exhibited the lowest proportion of heterozygotes (H_o_ = 0.426) and allelic richness (*A*_*R*_ = 10.2), and the highest inbreeding (*F*_*ic*_ = 0.474), whereas the opposite extreme values were found in the easternmost (Ci: *H*_*o*_ = 0.692, *A*_*R*_ = 16.7, *F*_*ic*_ = 0.245) and southernmost (Si: *H*_*o*_ = 0.719, *A*_*R*_ = 16.2, *F*_*ic*_ = 0.140) populations. However, only the Pa population differed significantly from the other ones, except for the Al population for *A*_*R*_.

**Table 2:** Genetic diversity and inbreeding statistics of the different cohorts and provenances, according to nine new microsatellite markers developed for this study.

Population	*N*^*a*^	*NA*_*E*_^*b*^	*A*_*R*_^*c*^ *(k*^*d*^*)*	*He*^*e*^	*Ho*^*f*^	*F*^*g*^	*Fic*^*h*^ *(95% posterior distribution)*
All populations (trees and progenies)	1249	12.82	26.84 (*k* = 464)	0.905	0.481	0.468	
Mature trees	246	13.00	26.82 (*k* = 464)	0.912	0.620	0.320	0.317 (0.294–0.336)
Seedlings	1003	12.61	26.42 (*k* = 464)	0.902	0.446	0.505	0.442 (0.429–0.457)
Seeds from 4 mother trees (ENEF garden)	140	4.52	2.87 (*k* = 4)	0.772	0.286	0.630	
Provenance							
Ci							
Adults	68	11.72	16.72 (*k* = 56)	0.897	0.692	0.231	0.245 (0.207–0.284)
Progenies	235	8.6	13.79 (k = 56)	0.856	0.476	0.445	0.391 (0.361–0.419)
Se							
Adults	33	8.52	14.72 (*k* = 56)	0.856	0.645	0.249	0.247 (0.195–0.308)
Progenies	204	7.04	11.77 (*k* = 56)	0.829	0.419	0.496	0.411 (0.376–0.454)
Si							
Adults	30	10.37	16.12 (*k* = 56)	0.872	0.719	0.178	0.140 (0.080–0.193)
Progenies	186	8.45	12.80 (*k* = 56)	0.840	0.470	0.442	0.316 (0.268–0.366)
Al							
Adults	67	9.45	13.36 (*k* = 56)	0.879	0.627	0.288	0.298 (0.260–0.335)
Progenies	203	8.11	11.43 (*k* = 56)	0.860	0.459	0.467	0.416 (0.388–0.444)
Pa							
Adults	48	5.58	10.25 (*k* = 56)	0.808	0.426	0.475	0.474 (0.427–0.520)
Progenies	175	6.37	09.76 (*k* = 56)	0.835	0.399	0.524	0.482 (0.455–0.516)

^a^Sample size.

^b^Effective number of alleles.

^c^Standardized allelic richness (rarefaction).

^d^Number of gene copies used for resampling.

^e^Gene diversity corrected for sample size (expected heterozygosity).

^f^Observed heterozygosity.

^g^Inbreeding coefficient (i.e., apparent heterozygote deficit).

^h^Inbreeding coefficient corrected for null alleles and 95% highest posterior distribution (INEST estimates).

Similar patterns were found at the seedling stage, but with lower *H*_*o*_ and *A*_*R*_ and higher *F*_*ic*_ values than in adults. Paired *t*-tests between adults and seedlings, considering each locus as a replicate, showed a significant difference for both *H*_*o*_ (*P*-value < 0.05) and *F*_*i*_ (*P*-value < 0.05). The inbreeding level (*F*_*i*_ or the corrected estimates *F*_*ic*_) of seedlings is about twice as high as in adults, except in Pa, where the difference is much more limited (Table 2). Populations within the SRI cluster showed pairwise *F*_ST_ values ranging from 0.026 (Ci–Si) to 0.09 (Al–Pa), with an average of 0.058 (Table S5).

Demographic signatures of population decline were significant in all *P. elata* provenance populations from the SRI genetic cluster except Ci (Table 3). The intensity of the bottleneck, reflected by the absolute values of Z-scores, increased westward across the populations (Table 3).

**Table 3:** Estimated Z-scores values of M-ratio for bottleneck testing in five provenance populations of Pericopsis elata from the SRI genetic cluster, with *p*-values indicating the presence of a bottleneck (**p < 0.01; *p < 0.05; NS = not significant), using INEST.

Provenance (SRI)	Z-scores
Ci	−1.024^NS^
Se	−1.841*
Si	−2.067*
Al	−2.760**
Pa	−3.029**

More negative Z-scores indicate a stronger departure of the M-ratio from expectations for a constant-size population in the direction of the past demographic bottleneck.

### Selfing rate in *P. elata*

The selfing rate was estimated for a total of 54 mother trees, with the number of seedlings identified as selfed or outcrossed ranging from 10 to 21 per mother tree (Table S3). It ranged from 0.64 to 1, with a mean of 0.88 (SD = 0.10). Ten families (18.5%) consisted entirely of selfed seedlings, while nine families (16.7%) showed intermediate selfing rates (0.20 < s < 0.80). Using the NMπ direct approach, the selfing rate of progenies was estimated at 0.85. These estimates were obtained after excluding 82 progenies (out of 1004) for which paternity analysis did not robustly classify them as selfed or outcrossed. The coefficient of consanguinity of these excluded progenies (*F* = 0.37) was intermediate between those of outcrossed (*F* = 0.12) and selfed (*F* = 0.57), suggesting either that they are made of a balanced mixture of selfed and outcrossed progenies, or that they are mostly made of outcrossed progenies between related parents. Considering the latter scenario, treating all uncertain progenies as outcrossed led to a mean selfing rate of 0.81 ± 0.04 (Table S6).

The difference in selfing rate between mother trees appeared to be just significant (χ² = 71.900, df = 53, *p* = 0.043). The estimated selfing rate within each provenance ranged from 0.84 ± 0.10 to 0.92 ± 0.07 (Table 4), suggesting a consistently predominant selfing strategy across the entire SRI cluster. The mean selfing rates did not differ significantly among the five provenances (Kruskal-Wallis rank sum test: χ² = 6.973, df = 4, *p* = 0.137), nor did their variance (Levene’s test for homogeneity of variance: *p* = 0.514). The DBH of the mother tree, its longitude, the density of trees within a 100 m radius, and the mean heterozygosity of the mother tree did not explain variation in selfing rates (Table S4).

**Table 4:** Estimated values of primary (s, proportion of selfed seedlings) and secondary (s′, proportion of selfed adults) selfing rates and of the inbreeding depression (δ) in five provenance populations of Pericopsis elata from the SRI genetic cluster, ordered from East to West. s was estimated by paternity analysis of 54 seedling families, while s′ and δ were estimated from a simulation algorithm accounting for the differences in inbreeding levels between adults, selfed seedlings, and outcrossed seedlings.

Provenance	*s* (SE)	s′ [95% highest posterior distribution]	δ [95% highest posterior distribution]
SRI cluster			
Ci	0.86 (0.081)	0.33 [0.24–0.44]	0.89 [0.81–0.95]
Se	0.92 (0.091)	0.36 [0.12–0.61]	0.95 [0.86–0.99]
Si	0.86 (0.116)	0.10 [0–0.3]	0.97 [0.90–1]
Al	0.84 (0.099)	0.45 [0.31–0.60]	0.82 [0.66–0.93]
Pa	0.92 (0.067)	0.86 [0.69–0.96]	0.37 [0–0.81]
Mean (sd)	0.88 (0.096)	0.42 (0.28)	0.80 (0.25)
Eastern cluster			
DRC	0.54 (Angbonda et al. 2021)	0.17 [0.08–0.26]	0.87 [0.74–0.95]

### Inbreeding depression in *P. elata*

The decay of the values of the inbreeding coefficients between the seedling stage and the adult stage (Table 2), indicates that although most of the seeds resulted from self-fertilization, the latter contributed less to the adult generation than the outcrossed ones. Our ABC algorithm indicates that all the populations exhibited significant lifetime inbreeding depression, which ranged from δ = 0.37 for Pa (westernmost population) to 0.97 for Si (southernmost population), with an average in the SRI cluster of 0.80 ± 0.24 (mean ± sd) (Table 4). According to the posterior distributions of the δ estimates in the SRI cluster and the Biaro population from the eastern cluster, all populations but Pa displayed overlapping 95% posterior distribution ranges around δ = 0.9, indicating that the survival rate to adulthood of selfed seedlings is about an order of magnitude lower than for outcrossed seedlings (Table 4). By contrast, in Pa, the survival rate of selfed seedlings is reduced by a third compared to outcrossed ones. We obtained very similar results when we included the 82 progenies of uncertain origin, assuming that they were outcrossed (mean δ = 0.81, SD = 0.04; Table S6).

### Effect of the number of seeds per pod and seed position in pods on selfing rate

The selfing rates of the seeds collected at the ENEF garden on four mother trees ranged from 0.88 to 1 per mother, with a mean of 0.91 ± 0.06. There was no significant difference in selfing rates among the mother trees (χ² = 2.897, df = 3, *p* = 0.408), between single-seeded and four-seeded pods (χ² = 0.013, df = 1, *p* = 0.908), nor in relation to the position of a seed in the ovary within four-seeded pods (χ² = 0.509, df = 1, *p* = 0.475).

## Discussion

In this study, we used microsatellites (SSRs) to characterize genetic diversity, the mating system, and inbreeding depression in five *P. elata* populations across the SRI colonization pathway. We expected that range expansion would erode genetic diversity and inbreeding depression, increase inbreeding, and facilitate a shift toward higher selfing.

### Reduced allelic richness and signal of demographic bottleneck at colonization front

During range expansion, the genetic diversity in newly established populations is expected to decline due to founder effects (Excoffier et al. 2009), a pattern well established for species that underwent historical range shifts due to postglacial range expansion (Hewitt 2000). Accordingly, in *P. elata*, we observed across the SRI range a westward decline in standardized allelic richness and, to a lesser extent, observed heterozygosity, with the western‑front population Pa showing the lowest values, and the easternmost and southernmost populations (Ci and Si) showing the highest values (Table 2). Successive founder events explain the moderate differentiation observed between populations (*F*_ST_ = 0.026–0.090), which are highest between pairs including the population closest to the colonization front (Pa; Table S5). However, we did not observe significant differences in expected heterozygosity between eastern and western populations, contrary to the signal previously reported with less polymorphic SSR markers (Micheneau et al. 2011; Ranavat et al. 2025). This discrepancy could result from our choice of selecting highly polymorphic SSR markers for the present work, using a genome coming from the Pa population. Moreover, founder events result primarily in the loss of rare alleles, affecting allelic richness more strongly than heterozygosity (Nei et al. 1975), so allelic richness often reveals demographic signals even when heterozygosity differences are subtle (Spencer et al. 2000). The allelic richness decline pattern is consistent with expansion driven by multiple founder events from a refugium in northern Republic of Congo; populations that expanded into Cameroon lost alleles during serial colonization, a process that, combined with selfing, may have accelerated the loss of rare alleles. Bottleneck tests corroborate this interpretation; only the easternmost, putative refugial population lacked a bottleneck signal, whereas the other four populations showed increasing bottleneck signatures toward the West. Hence, our data support the scenario of a westward expansion of *P. elata* from the northern Republic of Congo into Cameroon, which probably occurred after the last glacial maximum according to a time-calibrated phylogeny of plastomes (Ranavat et al. 2025), confirming our hypothesis H1.

### Selfing rates of *P. elata* across range expansion and genetic clusters

We found that SRI populations of *P. elata* are predominantly selfing, with a mean within the range of 0.81–0.88 depending on how uncertain progenies were treated, whereas in the eastern cluster the species shows a mixed-mating system (Angbonda et al. 2021). The selfing rate difference between these two clusters is pronounced (0.84–0.92 in SRI and 0.54 in the eastern cluster). Although different sets of microsatellites were used in the two studies, because the set successfully used in the eastern cluster was not polymorphic enough in the SRI cluster to conduct paternity analysis, and the studies were conducted in different years, the observed shift in selfing rates between the eastern and SRI clusters should not be affected by these differences for the following reasons. First, the large majority of progenies could be identified as selfed or outcrossed with high confidence in both studies. Second, the contrast in selfing rate estimates obtained at the progeny level is consistent with the contrast in inbreeding levels and small-scale spatial genetic structuring of adults between the SRI and the eastern clusters (Ranavat et al. 2025).

Selfing at the seed or seedling stage has also been documented in other co-occurring African tropical trees but at much lower rates, such as in *Baillonella toxisperma* (0.30) (Duminil et al. 2016a) and *Erythrophleum suaveolens* (0.16) (Duminil et al. 2016b), and in some Australian *Eucalyptus* such as *E. gomphocephala* (0.24) (Bradbury and Krauss 2013), *E. cladocalyx* (0.42) (McDonald et al. 2003), *E. benthamii* (0.39) (Butcher et al. 2005) and *E. regnans (*0.26) (Griffin et al. 2019). However, predominantly selfing populations in tree species are exceptional (Dick et al. 2008; Goodwillie et al. 2005; Ward et al. 2005). This result contrasts with the global trend for long-lived species to be outcrossing while short-lived species tend to be predominantly selfing (Duminil et al. 2009; Moeller et al. 2017; Snell and Aarssen 2005).

Furthermore, we hypothesized (H2) that recent demographic events in the SRI cluster could have shifted the mating system of *P. elata*. Founder events and long‑distance seed dispersal can favor the establishment of individuals with greater capacity for self‑fertilization when mates or pollinators are limiting (Levin 2012; Makowski et al. 2024), producing higher selfing in newly founded populations and a longitudinal increase in selfing across the expansion pathway (Baker 1955; Pannell 2016). Increased selfing following expansion has been documented in several short-lived species (e.g., Griffin and Willi 2014; Koski et al. 2019) and small trees (Kennedy et al. 2021), but it is uncommon among tree species (Barrett and Harder 2017). Our hypothesis H2 assumed that the refugial or ancestral population at the base of the expansion would show a mixed-mating system (similar to the eastern cluster), with progressively higher selfing toward recently established western populations. However, our results do not support H2: the putative refugial population Ci already exhibited a very high selfing rate of ≈0.86 compared with ≈0.54 in the eastern cluster. Within the SRI cluster, there was no significant difference in mean selfing rates among populations and thus no east-to-west gradient. Similar findings have been reported for the short-lived species *Crithmum maritimum* (Latron et al. 2020), where multiple colonization events from diverse source populations were invoked to mitigate founder effects and reduce mate limitation (Latron et al. 2020). In the case of *P. elata*, however, the absence of a detectable shift may simply reflect that selfing rate was already very high in the ancestral SRI cluster, leaving little scope for further directional increase during recent expansion. The high level of selfing in our putative ancestral population could have been caused by some environmental conditions, such as mate limitation, lower density, and pollinator scarcity (Morgan et al. 2005; Peterson and Kay 2015) during repeated contraction of the population in refugia (Ranavat et al. 2025), which could have selected individuals with high selfing capacity. This interpretation is consistent with Shi et al. (2024) on *Primula*, who concluded that historical range contractions and postglacial recolonization can promote selfing via recolonization and demographic processes. Individual selfing rate variation occurs in both SRI and the eastern cluster, but the range differs: 0.64–1.00 in SRI versus 0.44–0.85 in the eastern cluster (Angbonda et al. 2024). The cause of this difference is unknown, but one possibility is that repeated range contractions have probably favored genotypes with high selfing capacity in the putative ancestral SRI.

The high selfing rate observed in the SRI does not seem to depend substantially on environmental factors because even under plantation conditions in an open garden, such as those in the ENEF garden, where *P. elata* trees fruit much earlier than in dense natural forests, the selfing rate was similar to that reported in natural populations of the SRI cluster. Moreover, selfing rate was not correlated to characteristics of the mother tree, such as its size (dbh), the density of conspecific adults in its surroundings, or its heterozygosity. Hence, the contrasted selfing rates observed between the eastern and SRI *P. elata* clusters most probably originate from genetic differences. Further work on floral morphology (e.g., size and position of male and female organs) and development (e.g., timing of anthers and stigma maturity) might explain why different genetic clusters differ in selfing rates, as found in other species, like the increase of homostyly in some *Primula* in regions affected by past glaciation (Shi et al. 2024), or floral display in *Mimulus ringens* (Christopher et al. 2021). Such insights could provide a foundation for selective breeding strategies.

Thus, our result shows that a predominantly selfing mating system is not restricted to short-lived species, *P. elata* being an example, albeit unique to the best of our knowledge, of a tropical long-lived species that can also evolve high selfing rates. Contrary to our expectation (H2), its range expansion did not produce a detectable change in the mating system.

### Persistence of high inbreeding depression in highly self-pollinated populations

Inbreeding depression (δ), defined here as 1 – (fitness of selfed individual/fitness of outcrossed individuals), is mainly attributed to the expression of partially or fully recessive deleterious alleles. These alleles should be purged by selection as selfing increases (Barrett and Charlesworth 1991; Glémin 2003), or the least deleterious ones could be fixed during range expansion following repeated founder events (Pujol et al. 2009), decreasing δ in both situations. Therefore, the global trend in flowering plants is for lower δ in predominantly selfing lineages relative to outcrossing or mixed‑mating ones (Husband and Schemske 1996; Winn et al. 2011). Given the contrasting selfing rates between *P. elata* SRI and eastern populations, we expected weaker δ in predominantly selfing populations of *P. elata*. Using a Bayesian algorithm, we estimated δ across populations in the SRI and eastern clusters. Contrary to our hypothesis H3, we found very high δ in most populations of both clusters (≈0.82–0.97), except for Pa, a population at the expansion front that showed moderate inbreeding depression (≈0.37). The estimate for Pa was imprecise (95% posterior interval ≈0–0.81). These values imply that survival of selfed juveniles to adulthood is reduced by roughly 90% relative to outcrossed juveniles in all populations except Pa.

Previous work conducted in the eastern *P. elata* cluster reported a growth rate advantage of outcrossed over selfed seedlings that appears early and is stronger under low competition, when environmental conditions are optimal for growth (Angbonda et al. 2024). Functional traits of leaves did not differ between selfed and outcrossed seedlings, but increased phenotypic plasticity in outcrossed ones for some traits highlighted a potential mechanism through which inbreeding depression could occur (Ngongo et al. 2025). Angbonda et al. (2024) reported a lifetime inbreeding depression for the eastern cluster (Biaro population) of δ = 0.70, while we found δ = 0.87 [0.74–0.95] using the same data, but a different method. The approach used by Angbonda et al. (2024) was based on the decay in selfing rate between seedlings (*s* = 0.54) and adults (*s*′ = 0.26), where *s*′ was estimated from identity disequilibrium, while the approach used here is based on the decay of the inbreeding coefficient between cohorts, given the observed levels of inbreeding of selfed and outcrossed seedlings. Both methods rely on two main assumptions. (i) The primary selfing rate did not change over time: we assume that the selfing rate observed in our studied seedlings is representative of the rate occurring among seedlings when the studied adult trees were born. (ii) Inbreeding depression affects only selfed individuals: the effect of biparental inbreeding on relative fitness is ignored. However, the method used in the present study is expected to be more robust because the impact of biparental inbreeding on outcrossed seedlings is taken into account through marker-based inbreeding estimation of outcrossed seedlings, while the indirect estimation of *s*′ in Angbonda et al. (2024) could be overestimated due to biparental inbreeding. Accordingly, our Bayesian algorithm inferred that *s*′ = 0.17 [0.08–0.26] in the eastern cluster, an estimate lower than the one deduced from identity disequilibrium.

The maintenance of high inbreeding depression despite moderate or predominant selfing has been reported in several other species (e.g., Christopher et al. 2021; Delmas et al. 2014; Ishida 2006; Michalski and Durka 2007; Tamaki et al. 2009). Theory predicts that δ > 0.5 should prevent the evolution of predominant selfing (Goodwillie et al. 2005), yet *P. elata* exhibits both high selfing and high inbreeding depression. Classical purging theory (Barrett and Charlesworth 1991; Glémin 2003; Pujol et al. 2009) has difficulty explaining this persistence because long-term selfing should remove strongly deleterious recessive alleles. Lande et al. (1994) proposed that this apparent contradiction can be reconciled if (i) the genomic mutation rate for recessive deleterious alleles is sufficiently high and (ii) selective interference among loci impedes purging. These mechanisms can inhibit effective purging even in predominantly selfing populations, particularly in large, long-lived plants that may accrue greater per-generation mutation loads (Scofield and Schultz 2006). Another potential explanation is pseudo-overdominance, which can occur when different sets of (partially) recessive deleterious mutations occur in different haplotypes of a non-recombining genomic region, so that heterozygotes for these haplotypes have higher fitness than homozygotes, causing a balancing selection where deleterious alleles cannot be purged unless recombination occurs (Abu-Awad and Waller 2023). Under such a scenario, high genetic drift could nevertheless fix one haplotype, reducing inbreeding depression because there is no more opportunity to benefit from a heterosis effect. This would be compatible with the observation of a reduction of δ limited to the colonization front (population Pa), independent of the selfing rate. This may be a transient situation, as occasional long-distance pollen flow might bring back new haplotypes that would be selected for in heterozygotes and restore high inbreeding depression. Analyses of mutation load and signatures of pseudo-overdominance will be essential to elucidate the genetic architecture of inbreeding depression and to evaluate evidence for purging of deleterious mutations in *P. elata*.

### No effect of seed set per pod or within‑pod position on selfing rate

Sourcing seeds that are highly sensitive to inbreeding depression can reduce plantation performance (Costa e Silva et al. 2010) and long-term persistence (Griffin et al. 2019). Inbreeding depression has been shown to negatively affect plant growth in numerous species (e.g., Ismail et al. 2014; Naito et al. 2005; Rymer et al. 2015), including *P. elata* (Angbonda et al. 2024; Ngongo et al. 2025). If a population exhibits substantial selfing, random collection of seed lots risks increasing the proportion of selfed progeny in plantations and thereby reducing vigor over time. In some taxa like *Medicago sativa*, seed number per pod and seed position within multiseeded pods sometimes correlate with mating origin because of early zygotic abortion and differential pollen‑tube growth (Cooper and Brink 1940; Horovitz et al. 1976; Pedersen 1968). When such patterns are strong and well‑validated, they offer a low‑cost method to bias collections toward outcrossed progeny in contexts where molecular screening is impractical. However, in *P. elata*, we found no evidence that these proxies are informative: selfing rates did not differ between pods with one versus four seeds (*p* = 0.908), and both selfed and outcrossed seeds occurred randomly within multiseeded pods (*p* = 0.475), refuting our hypothesis H4. Hence, we cannot propose a strategy to obtain higher-quality outcrossed seeds for plantations, except using direct genetic assessment. Nevertheless, in this study as well as in Angbonda et al. (2024), moderate albeit statistically significant differences in selfing rates were observed among adult trees. It is unknown if these intra-cluster differences have a genetic and/or environmental basis, but if they could be correlated to floral morphological traits, selective breeding strategies favoring outcrossers could be based on floral traits.

## Conclusion

This study aimed to analyze the mating system of *Pericopsis elata*, a tropical tree species with a fragmented distribution in Central Africa. Our results revealed a shift toward increased self-fertilization in the SRI cluster compared to the eastern cluster. This shift could be linked to recent demographic events in the SRI cluster. Populations seem to have expanded from the northern Republic of Congo to Cameroon. However, this expansion does not appear to have influenced the selfing rate within the SRI cluster, as all populations located along the recolonization pathway presented similarly high selfing rates. Furthermore, SRI populations exhibited variation in inbreeding depression (ID), with lower ID found in the most recently established population. This pattern aligns with purging theory, where serial founder events could reduce deleterious alleles. Nevertheless, despite major differences in selfing rates between the two clusters, they showed only weak differences in ID, suggesting that purging may not have been as effective as theoretically expected. Additionally, no correlation was observed between pod characteristics, such as the number of seeds per pod or their position, and whether seeds resulted from selfing or cross-fertilization. The distinct selfing rates observed in the two main clusters of *P. elata* in Central Africa highlight the importance of considering these differences when developing conservation strategies for the species. Moreover, despite consistently high selfing rates, populations within the SRI cluster exhibit severe lifetime inbreeding depression, raising a fundamental paradox. Classical theoretical predictions suggest that increased selfing should facilitate the purging of deleterious mutations, thereby reducing inbreeding depression. These findings provide new insights into the evolution of mating systems in tropical plant species.

## Supplementary information

Supplementary materials

## Data Availability

All genotypes and the R routine are available from 10.5281/zenodo.17572330.
