# Supplementary material for: High selfing rate and inbreeding depression correlate with past range expansion in an endangered African timber species Pericopsis elata
Source: Heredity (Edinb). 2026 Apr 23;135(7):465–75. doi: 10.1038/s41437-026-00845-6 (PMC13451301; doi:10.1038/s41437-026-00845-6)
Supplement: Supplementary file 1 [file 41437_2026_845_MOESM1_ESM.docx]

**High selfing rate and inbreeding depression correlate with past range expansion in an endangered African timber species *Pericopsis elata***

**Supplementary materials**

**S1: PCR protocol and** characteristics of nine new microsatellite primers developed for *Pericopsis elata*

New SSR loci were searched manually from (i) Illumina reads of a genomic library sequenced on a NextSeq device (sample RP0242 collected in Cameroon), (ii) a preliminary draft genome of *P. elata* obtained after sequencing a seedling from the Pa population using an Oxford Nanopore MinION device (S. Ranavat unpublished results). We searched for long SSRs with at least 15 or 30 AT repeats in the genomic library and draft genome, respectively. After selecting 24 loci for which forward and reverse primers could be designed according to Primer3 (Koressaar & Remm 2007), we ordered forward and reverse primers, adding one of four tails (Q1, Q2, Q3, Q4) to the 5’ end of the forward primers to mark the amplicons with a fluorescent dye, as explained in Micheneau et al. (2011). Amplification tests were performed on multiplexes of four loci (one per distinct dye) on eight samples, and marker polymorphism and readability were assessed on a 48-capillary ABI 3730 sequencer, using the Liz500 size standard and the following dyes: 6-FAM (for Q1), NED (for Q2), VIC (for Q3), or PET (for Q4). Nine loci were considered both highly polymorphic and easily interpretable (P76, P78, P90, P91, P92, P93, P95, P96, P97) for routine analyses. Each PCR reaction contained 1.5 μL of extracted DNA, with final primer concentrations of 0.07 μM for each forward primer, 0.2 μM for each reverse primer and each of the four fluorescently labeled primers (Q1 to Q4). Thermal cycling conditions were as follows: an initial denaturation at 95 °C for 15 minutes; followed by 23 cycles of denaturation at 94 °C for 30 seconds, annealing at 57 °C for 90 seconds, and extension at 72 °C for 90 seconds; then 10 additional cycles of denaturation at 94 °C for 30 seconds, annealing at 53 °C for 30 seconds, and extension at 72 °C for 45 seconds; with a final extension at 60 °C for 30 minutes.

**Table S1:** Characterization of 9 microsatellite markers isolated from *Pericopsis elata*. Forward and reverse sequences, labeled primer, repeat motif, range of allele sizes (bp) and PCR product size (bp) are shown for each primer pair.

| Locus | Primer sequence (5’-3’)* | Labeled primer* | Repeat motif | Allele size (bp) | PCR product size (bp) |
| --- | --- | --- | --- | --- | --- |
| P76‡ | F: Q4- CTAGTTATTGCTCAGCGGTAGACACTTGTATGTAAGCATGGA  R: CGTTAGATTACAATGAAGATTTTTGCA | Q4-PET | (AT)_27_ | 108-182 | 155 |
| P78† | F: Q3- CACTGCTTAGAGCGATGCAGTAAAATTTATTTGGTGCTTGTGATT  R: ATCTATGGTCAATTTAGAAGAGAGGA | Q3-VIC | (AT)_17_ | 127-191 | 126 |
| P90‡ | F: Q1- CACTGCTTAGAGCGATGCACCTAATGATGGTGTATGGGTGT  R: ACCTAGTACTTGACTAAGATAGGGGT | Q1-6-FAM | (AT)_30_ | 171-257 | 193 |
| P91† | F: Q1- TGTAAAACGACGGCCAGTAGAACTTTGTTTTCCCTTCATTTTGA  R: CCATCAAACCTATAAGTCTTACGAAGG | Q1-6-FAM | (AT)_30_ | 161-226 | 176 |
| P92‡ | F: Q3- CACTGCTTAGAGCGATGCACGGGAATTGCCGTACTTCT  R: ACTAAACTCAGTGTCCAACATGAA | Q3-VIC | (AT)_30_ | 112-184 | 143 |
| P93† | F: Q2-TAGGAGTGCAGCAAGCATTGGCTTGTTTTACTTATTTTTGATGGA  R: AAGTGTTTGCCATAACTCCTTTCA | Q2-NED | (AT)_30_ | 153-209 | 175 |
| P95‡ | F: Q3-CACTGCTTAGAGCGATGCAGTGATCGCCTACTGATTGAGT  R: ACTGCAAGATATCCACAAATTATTCA | Q3- VIC | (AT)_30_ | 186-250 | 206 |
| P96‡ | F: Q2- TAGGAGTGCAGCAAGCATTGAAGGAGGATACACTGGACA  R: AGAGGGATTACATGGCCTAAGT | Q2-NED | (AT)_30_ | 154-221 | 177 |
| P97† | F: Q4- CTAGTTATTGCTCAGCGGTAGGCCCTTCACCTTATGCTT  R: ATGAAGCCGATGGAACCTCC | Q4-PET | (AT)_30_ | 150-204 | 168 |
|  |  |  |  |  |  |
|  |  |  |  |  |  |

Notes. * Q1: TGTAAAACGACGGCCAGT; Q2: TAGGAGTGCAGCAAGCAT ; Q3: CACTGCTTAGAGCGATGC ; Q4: CTAGTTATTGCTCAGCGGT. † and ‡ Locus included in multiplex 11C and 12C, respectively.

**Table S2.** Genetic characterization of nine newly developed microsatellite markers in adult trees from five populations of Pericopsis elata

|  | | Ci *(N=68)* | | | | Si *(N=30)* | | | | Se *(N=33)* | | | | Al *(N=67)* | | | | Pa *(N=48)* | | | |
| --- | --- | --- | --- | --- | --- | --- | --- | --- | --- | --- | --- | --- | --- | --- | --- | --- | --- | --- | --- | --- | --- |
| Locus | *Na_t_* | *Na* | *H_e_* | *H_o_* | *F_i_* | *Na* | *H_e_* | *H_o_* | *F_i_* | *Na* | *H_e_* | *H_o_* | *F_i_* | *Na* | *H_e_* | *H_o_* | *F_i_* | *Na* | *H_e_* | *H_o_* | *F_i_* |
| P76 | 25 | 22 | 0.928 | 0.603 | 0.352 | 18 | 0.930 | 0.833 | 0.105 | 17 | 0.921 | 0.677 | 0.268 | 19 | 0.912 | 0.477 | 0.479 | 10 | 0.802 | 0.372 | 0.539 |
| P78 | 26 | 17 | 0.866 | 0.667 | 0.232 | 17 | 0.918 | 0.714 | 0.225 | 16 | 0.817 | 0.636 | 0.224 | 15 | 0.907 | 0.727 | 0.199 | 17 | 0.851 | 0.375 | 0.562 |
| P90 | 38 | 26 | 0.946 | 0.809 | 0.146 | 18 | 0.938 | 0.833 | 0.113 | 22 | 0.934 | 0.727 | 0.224 | 25 | 0.935 | 0.714 | 0.238 | 13 | 0.883 | 0.489 | 0.448 |
| P91 | 21 | 15 | 0.867 | 0.667 | 0.232 | 16 | 0.903 | 0.733 | 0.191 | 13 | 0.871 | 0.645 | 0.262 | 13 | 0.861 | 0.563 | 0.348 | 10 | 0.738 | 0.439 | 0.408 |
| P92 | 29 | 27 | 0.947 | 0.731 | 0.229 | 17 | 0.883 | 0.767 | 0.133 | 19 | 0.917 | 0.727 | 0.209 | 19 | 0.912 | 0.776 | 0.150 | 10 | 0.811 | 0.438 | 0.463 |
| P93 | 24 | 14 | 0.781 | 0.618 | 0.210 | 10 | 0.622 | 0.500 | 0.199 | 12 | 0.830 | 0.606 | 0.273 | 13 | 0.859 | 0.582 | 0.324 | 12 | 0.800 | 0.383 | 0.524 |
| P95 | 26 | 22 | 0.918 | 0.723 | 0.214 | 17 | 0.932 | 0.724 | 0.226 | 16 | 0.897 | 0.636 | 0.294 | 16 | 0.896 | 0.591 | 0.342 | 11 | 0.735 | 0.444 | 0.398 |
| P96 | 29 | 25 | 0.911 | 0.687 | 0.248 | 19 | 0.894 | 0.733 | 0.179 | 13 | 0.779 | 0.576 | 0.264 | 13 | 0.880 | 0.687 | 0.221 | 10 | 0.786 | 0.458 | 0.420 |
| P97 | 24 | 18 | 0.912 | 0.721 | 0.211 | 16 | 0.836 | 0.633 | 0.245 | 12 | 0.736 | 0.576 | 0.220 | 11 | 0.747 | 0.530 | 0.291 | 13 | 0.866 | 0.438 | 0.497 |
| Mean | 26.9 | 20.7 | 0.892 | 0.691 | 0.230 | 16.4 | 0.872 | 0.719 | 0.180 | 15.6 | 0.856 | 0.645 | 0.249 | 16 | 0.879 | 0.627 | 0.288 | 11.8 | 0.808 | 0.426 | 0.473 |

*Na* = number of alleles; *Na_t_* = total numbers of alleles observed among all five populations; *F_i_* = Inbreeding coefficient; *H_e_* = expected heterozygosity; *H_o_* = observed heterozygosity; *N* = number of individuals

**Table S3.** Estimation of selfing rate per family

| Prov | Seed  tree | Lon | Lat | Diameter (cm) | Total_juv  assigned | Nb_juv  outcrossed | Nb_juv  selfed | Selfing  rate |
| --- | --- | --- | --- | --- | --- | --- | --- | --- |
| Al | Al04 | 14.714 | 3.133 | 90.0 | 19 | 2 | 17 | 0.895 |
| Al | Al16 | 14.715 | 3.135 | 89.4 | 17 | 3 | 14 | 0.824 |
| Al | Al23 | 14.721 | 3.135 | 80.0 | 17 | 4 | 13 | 0.765 |
| Al | Al25 | 14.721 | 3.130 | 90.3 | 14 | 2 | 12 | 0.857 |
| Al | Al29 | 14.715 | 3.133 | 85.0 | 14 | 1 | 13 | 0.929 |
| Al | Al38 | 14.702 | 3.096 | 95.0 | 14 | 5 | 09 | 0.643 |
| Al | Al44 | 14.701 | 3.122 | 98.2 | 15 | 4 | 11 | 0.733 |
| Al | Al48 | 14.718 | 3.109 | 127.3 | 18 | 3 | 15 | 0.833 |
| Al | Al52 | 14.703 | 3.096 | 89.5 | 18 | 0 | 18 | 1.000 |
| Al | Al66 | 14.698 | 3.133 | 91.1 | 18 | 2 | 16 | 0.889 |
| Al | Al79 | 14.702 | 3.140 | 133.2 | 19 | 2 | 17 | 0.895 |
| Ci | Ci02 | 16.118 | 2.055 | 84.0 | 19 | 3 | 16 | 0.842 |
| Ci | Ci06 | 16.121 | 2.053 | 115.5 | 18 | 1 | 17 | 0.944 |
| Ci | Ci19 | 16.141 | 2.038 | 105.2 | 20 | 3 | 17 | 0.850 |
| Ci | Ci21 | 16.132 | 2.038 | 73.7 | 21 | 2 | 19 | 0.905 |
| Ci | Ci32 | 16.105 | 2.081 | 106.5 | 14 | 4 | 10 | 0.714 |
| Ci | Ci36 | 16.099 | 2.067 | 106.8 | 13 | 2 | 11 | 0.846 |
| Ci | Ci41 | 16.096 | 2.071 | 122.2 | 19 | 1 | 18 | 0.947 |
| Ci | Ci51 | 16.103 | 2.009 | 159.4 | 20 | 1 | 19 | 0.950 |
| Ci | Ci59 | 16.106 | 2.006 | 89.6 | 18 | 2 | 16 | 0.889 |
| Ci | Ci62 | 16.117 | 1.998 | 78.9 | 17 | 3 | 14 | 0.824 |
| Ci | Ci71 | 16.109 | 2.003 | 98.9 | 15 | 4 | 11 | 0.733 |
| Pa | Pa10 | 14.017 | 3.404 | 41.3 | 15 | 3 | 12 | 0.800 |
| Pa | Pa12 | 14.031 | 3.398 | 95.7 | 17 | 0 | 17 | 1.000 |
| Pa | Pa16 | 14.220 | 3.172 | 88.8 | 18 | 3 | 15 | 0.833 |
| Pa | Pa33 | 13.997 | 3.432 | 96.9 | 20 | 1 | 19 | 0.950 |
| Pa | Pa35 | 14.021 | 3.418 | 69.4 | 16 | 1 | 15 | 0.938 |
| Pa | Pa36 | 14.025 | 3.417 | 84.1 | 14 | 1 | 13 | 0.929 |
| Pa | Pa40 | 14.024 | 3.418 | 87.1 | 17 | 1 | 16 | 0.941 |
| Pa | Pa46 | 14.027 | 3.421 | 85.4 | 15 | 1 | 14 | 0.933 |
| Pa | Pa51 | 14.014 | 3.405 | 111.9 | 10 | 0 | 10 | 1.000 |
| Se | Se02 | 15.848 | 2.570 | 65.5 | 15 | 1 | 14 | 0.933 |
| Se | Se03 | 15.849 | 2.569 | 91.5 | 17 | 3 | 14 | 0.824 |
| Se | Se10 | 15.841 | 2.555 | 100.0 | 17 | 2 | 15 | 0.882 |
| Se | Se26 | 15.829 | 2.575 | 76.5 | 16 | 0 | 16 | 1.000 |
| Se | Se29 | 15.827 | 2.573 | 62.2 | 18 | 1 | 17 | 0.944 |
| Se | Se30 | 15.828 | 2.576 | 112.4 | 19 | 0 | 19 | 1.000 |
| Se | Se33 | 15.828 | 2.582 | 113.5 | 12 | 2 | 10 | 0.833 |
| Se | Se34 | 15.826 | 2.580 | 123.0 | 18 | 5 | 13 | 0.722 |
| Se | Se35 | 15.829 | 2.580 | 97.7 | 11 | 1 | 10 | 0.909 |
| Se | Se36 | 15.830 | 2.579 | 98.2 | 19 | 0 | 19 | 1.000 |
| Se | Se41 | 15.922 | 2.698 | 69.8 | 15 | 0 | 15 | 1.000 |
| Se | Se48 | 15.805 | 2.718 | 75.7 | 15 | 0 | 15 | 1.000 |
| Si | Si02 | 15.235 | 1.769 | 59.9 | 16 | 0 | 16 | 1.000 |
| Si | Si07 | 15.320 | 1.928 | 66.2 | 14 | 5 | 09 | 0.643 |
| Si | Si08 | 15.290 | 1.958 | 73.7 | 15 | 3 | 12 | 0.800 |
| Si | Si14 | 15.291 | 1.968 | 51.5 | 18 | 2 | 16 | 0.889 |
| Si | Si18 | 15.292 | 1.970 | 103.6 | 14 | 1 | 13 | 0.929 |
| Si | Si19 | 15.287 | 1.944 | 75.3 | 16 | 4 | 12 | 0.750 |
| Si | Si21 | 15.278 | 1.922 | 60.0 | 20 | 1 | 19 | 0.950 |
| Si | Si25 | 15.293 | 1.941 | 67.6 | 10 | 0 | 10 | 1.000 |
| Si | Si30 | 15.347 | 1.890 | 65.3 | 14 | 2 | 12 | 0.857 |
| Si | Si35 | 15.283 | 1.942 | 111.5 | 20 | 2 | 18 | 0.900 |
| Si | Si37 | 15.273 | 1.943 | 73.4 | 11 | 3 | 08 | 0.727 |

**Table S4.** Modelling of the estimated primary selfing rate per mother tree according to its diameter at breast height, longitude, local density of adult trees within 100 m, and observed heterozygosity (Ho), using a Generalized Linear Model.

| Predictor | Estimate (SE) | Z value | P-value |
| --- | --- | --- | --- |
| Intercept | 2.495 (9.354) | 0.267 | 0.790 |
| Diameter | -0.003 (0.020) | -0.135 | 0.893 |
| Longitude | -0.067 (0.616) | -0.109 | 0.913 |
| Local density  (100 m radius) | -0.015 (0.178) | -0.086 | 0.932 |
| Ho | 1.107 (2.312) | 0.479 | 0.632 |

**Table S5.** Pairwise genetic differentiation index *(F*_ST_*)* of the Sangha River Interval (SRI) populations of *Pericopsis elata* in Central Africa based on 9 microsatellite markers.

| Populations | Ci | Se | Si | Al | Pa |
| --- | --- | --- | --- | --- | --- |
| Ci |  | 0.034 | 0.026 | 0.053 | 0.065 |
| Se |  |  | 0.030 | 0.064 | 0.076 |
| Si |  |  |  | 0.063 | 0.080 |
| Al |  |  |  |  | 0.090 |
| Pa |  |  |  |  |  |

**Table S6.** Estimated values of primary (s, proportion of selfed seedlings) and secondary (s’, proportion of selfed adults) selfing rates and of the inbreeding depression (δ) in five provenance populations of Pericopsis elata from the SRI genetic cluster, ordered from East to West. s was estimated by paternity analysis of 54 seedling families, while s’ and δ were estimated from a simulation algorithm accounting for the differences in inbreeding levels between adults, selfed seedlings and outcrossed seedlings. Here, 82 progenies for which the paternity test could not confidently classify individuals as selfed or outcrossed (P < 0.8) were treated as outcrossed.

| Provenance | *s* (SE) | s’ [95% highest posterior distribution] | δ [95% highest posterior distribution] |
| --- | --- | --- | --- |
| Ci | 0.77 (0.16) | 0.24 [0.12-0.37] | 0.89 [0.77-0.96] |
| Se | 0.86 (0.12) | 0.21 [0-0.52] | 0.95 [0.83-1] |
| Si | 0.78 (0.11) | 0.06 [0-0.23] | 0.97 [0.89-1] |
| Al | 0.80 (0.11) | 0.32 [0.15-0.51] | 0.86 [0.68-0.96] |
| Pa | 0.83 (0.16) | 0.70 [0.35-0.88] | 0.40 [0-0.88] |
| Mean (sd) | 0.81 (0.04) | 0.31 (0.24) | 0.81 (0.23) |
